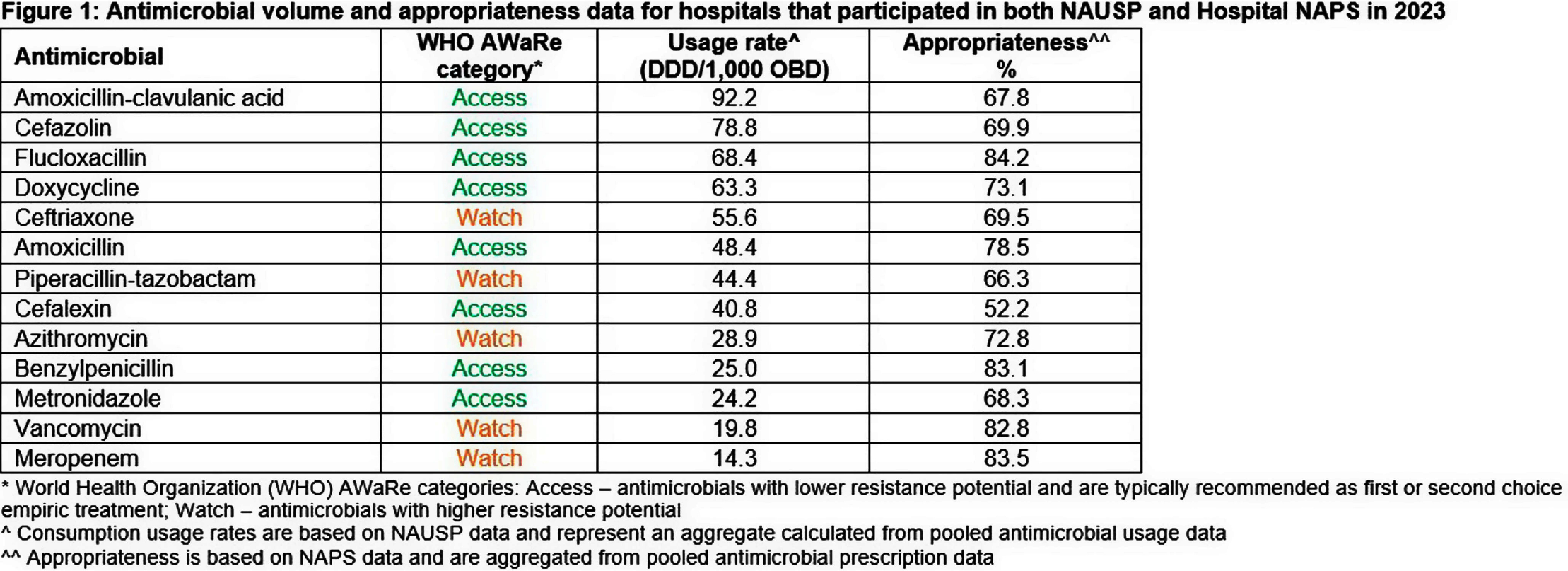# Antimicrobial Consumption and Appropriateness in Australian Hospitals: a Parallel Analysis of 2023 National Surveillance Data

**DOI:** 10.1017/ash.2025.219

**Published:** 2025-09-24

**Authors:** Caroline Chen, Erin Connor, Josephine Wen, Karin Thursky, Rodney James

**Affiliations:** 1 National Centre for Antimicrobial Stewardship; The Royal Melbourne Hospital; 2 University of Melbourne; 3 National Centre for Antimicrobial Stewardship

## Abstract

**Background:** A comprehensive understanding of antimicrobial prescribing practices, requires antimicrobial stewardship (AMS) clinicians to assess both the quantity and quality of antimicrobial prescribing. In Australia, two national programs collect and analyse such data in the hospital setting; the National Antimicrobial Utilisation Surveillance Program (NAUSP) a continuous, volume-based surveillance program that monitors antimicrobial usage trends; and the Hospital National Antimicrobial Prescribing Survey (Hospital NAPS) a standardised auditing program that assesses antimicrobial prescribing appropriateness. This study aims to analyse the 2023 NAUSP and Hospital NAPS data to compare the volume and appropriateness of inpatient antimicrobial use in Australian hospitals. **Methods:** Data were extracted from hospitals that participated in both programs in 2023, including systemically administered antimicrobials for adult patients. NAUSP data were aggregated and acute inpatient usage rates relative to patient activity were calculated as Defined Daily Doses (DDD) per 1,000 Occupied Bed Days (OBD) for individual antimicrobials.

Hospital NAPS data on appropriateness of prescribing, as assessed by local auditors as a point prevalence survey and using a standardised assessment matrix, were aggregated and calculated for each antimicrobial. Antimicrobials with high-volume use (NAUSP data), high rates of antimicrobial prescribing (NAPS data), or classified as medium to high risk for antimicrobial resistance potential according to the World Health Organization AWaRe classifications were further investigated. **Results:** There were 192 acute care hospitals that contributed in 2023 to both NAUSP (representing 12, 619, 253 OBDs) and Hospital NAPS (representing 21,017 antimicrobial prescriptions). Figure 1 summarizes the aggregate usage rates and appropriateness for antimicrobials of interest. Antimicrobials with the highest usage rates, including amoxicillin-clavulanic acid and cefazolin (92.2 and 78.8 DDD/1,000 OBD respectively), had moderate levels of appropriateness (67.8% and 69.9% respectively). Similar ‘Access‘ antimicrobials such as doxycycline, amoxicillin and metronidazole, which tend to be unrestricted in hospital formularies, had moderate levels of appropriateness. Cefalexin had the lowest rate of appropriateness (52.2%). In comparison, ‘Watch‘ antimicrobials, including meropenem and vancomycin, which tend to be restricted, had lower usage rates (14.3 and 19.8 DDDs/1,000 OBD respectively) but higher rates of appropriateness (83.5%, 82.8% respectively). **Conclusion:** This analysis highlights the importance of assessing and analyzing antimicrobial quantity and quality concurrently, providing a holistic view of prescribing practices. Furthermore, AMS efforts should include all antimicrobials, regardless of restriction category, as commonly used, unrestricted antimicrobials may have substantial rates of inappropriate prescribing.

**Figure:**